# Advanced Glycation End-Products in Common Non-Infectious Liver Diseases: Systematic Review and Meta-Analysis

**DOI:** 10.3390/nu13103370

**Published:** 2021-09-25

**Authors:** Kamil Litwinowicz, Ewa Waszczuk, Andrzej Gamian

**Affiliations:** 1 Department of Biochemistry and Immunochemistry, Wroclaw Medical University, 50-368 Wrocław, Poland; 2 Department of Gastroenterology and Hepatology, Wroclaw Medical University, 50-556 Wrocław, Poland; ewa.waszczuk@umed.wroc.pl; 3 Hirszfeld Institute of Immunology and Experimental Therapy, Polish Academy of Sciences, 53-114 Wrocław, Poland; andrzej.gamian@hirszfeld.pl

**Keywords:** serum levels of AGE, advanced glycation end-products, liver disease, alcohol, fructose

## Abstract

Background: Excessive intake of fructose, glucose and alcohol is associated with the development of non-alcoholic fatty liver disease (NAFLD) and alcoholic liver disease (ALD). At the same time, these dietetic factors create an environment favorable for the generation of advanced glycation end-products. For this reason, advanced glycation end-products (AGEs) are hypothesized to play role in the development of NAFLD and ALD. In this systematic review and meta-analysis, we explore the relationship between NAFLD and ALD with AGE levels, including their diagnostic accuracy. Methods: The systematic review and meta-analysis has been pre-registered with PROSPERO (CRD42021240954) and was performed in accordance with the PRISMA guidelines. Meta-analyses were performed using the meta R package. Results: We have obtained 11 studies meeting our inclusion criteria, reporting data on 1844 participants (909 with NAFLD, 169 with ALD and 766 healthy controls). NAFLD was associated with significantly higher AGE fluorescence and serum N-(carboxyethyl)lysine (CEL) levels. Patients with alcoholic cirrhosis had significantly higher levels of N-(carboxymethyl)lysine (CML). Only individual studies examined AGEs in the context of their diagnostic accuracy. AGE fluorescence distinguished low and moderate steatosis with an AUC of 0.76. The ratio of CML, CEL and pentosidine to a soluble variant of the AGE receptor differentiated patients with NAFLD from healthy controls with high AUC (0.83–0.85). Glyceraldehyde-derived AGE separated non-alcoholic fatty liver (NAFL) from non-alcoholic steatohepatitis (NASH) with acceptable performance (AUC 0.78). Conclusions: In conclusion, NAFLD and ALD are associated with significantly higher levels of several AGEs. More research is needed to examine the diagnostic accuracy of AGEs, however individual studies show that AGEs perform well in distinguishing NAFL from NASH.

## 1. Introduction

Chronic liver diseases account for over 2% of the global total deaths [1]. The percentage contribution of different etiologies is constantly shifting, with non-alcoholic fatty liver disease (NAFLD) and alcoholic liver disease (ALD) increasing in incidence worldwide. NAFLD is estimated to affect 24% of the global population and is currently one of the main causes of the increase in the incidence of cirrhosis [2]. ALD is responsible for 27% of deaths related to liver disease and 30% of cases of liver cancer worldwide [3].

NAFLD is defined as the presence of at least 5% hepatic steatosis in biopsy specimens. It includes non-alcoholic fatty liver (NAFL) and non-alcoholic steatohepatitis (NASH). Currently, the only diagnostic tool which reliably differentiates NAFL from NASH is a biopsy. In the case of NAFL, a biopsy must show one of the following appearances: (1) simple steatosis; (2) steatosis with lobular/portal inflammation, without ballooning; (3) steatosis with ballooning but without inflammation. Diagnosis of NASH requires steatosis with both ballooning and lobular inflammation [4]. NAFLD commonly coexists with insulin resistance (IR) and other disorders associated with it, including metabolic syndrome (MetS) [5]. The lifestyle risk factors of NAFLD are similar to those of MetS and include obesity, excessive intake of saturated fats, refined carbohydrates and fructose [6].

ALD refers to liver damage caused by excessive intake of alcohol (typically defined as regular consumption of >30 g of ethanol a day for males and >20 g for females). Analogous to NAFLD, it encompasses a wide range of disease severity, including steatosis, steatohepatitis (ASH) and cirrhosis (AC). Even though biopsy remains the mainstay of the ASH diagnosis, the American Association for the Study of Liver Diseases states that probable diagnosis can be established using clinical criteria [7]. The risk factors for the development and progression of ALD consist of various lifestyle (for example, drinking patterns—daily heavy drinking results in greater risk than binge drinking [8]) and genetic factors (e.g., disturbances in circadian clock genes [9,10]).

Although NAFLD and ALD are caused by different factors, they share similar clinical courses. The initial stage for both diseases is simple steatosis, which progresses further to steatohepatitis, fibrosis or cirrhosis in a relatively small fraction of patients with ALD and an even smaller proportion of patients with NAFLD. In the case of patients with alcohol use disorder (AUD), over 90% develop hepatic steatosis, 20–40% fibrosis and 8–20% cirrhosis [11]. In the case of NASH, progression to cirrhosis occurs in less than 2.5% of patients [12]. While multiple risk factors for the development and progression of NAFLD and ALD have been already identified, it is still challenging to identify patients at the highest risk of progression with high certainty and reliability.

Similarities in the clinical course are mirrored by multiple pathophysiological features shared between NAFLD and ALD. These include—but are not limited to—disturbances in circadian clock genes, alterations in the composition of the microbiome and increased intestinal permeability [9,13,14,15,16]. Recently advanced glycation end-products (AGEs) have been identified as an additional potential factor associated with the pathophysiology of both NAFLD and ALD [17].

AGEs are generated by non-enzymatic Maillard reactions between the carbonyl group of reducing sugar (or other carbonyl compounds) and a free amino group of a protein, aminophospholipid or nucleic acid. The first stage results in the generation of Schiff bases, which are then rearranged to stable Amadori products. Further oxidation and glycation result in the generation of AGEs [17,18,19]. Early research on AGEs focused mainly on the fluorescent fraction of AGEs; however, advances in biochemistry allowed detection of non-fluorescent fractions, with N-(carboxymethyl)lysine (CML) and pentosidine being the most widely studied. Novel approaches have allowed for the detection of even trace amounts of non-standard AGE subtypes [20]. 

Currently, the leading hypothesis for the involvement of AGEs in NAFLD and ALD is the toxic AGE (TAGE) theory, which focuses on the role of glyceraldehyde- and acetaldehyde-derived AGEs (GA- and AA-AGEs). GA and AA are produced in the liver through three pathways—fructolysis, glycolysis and alcoholysis. As increased consumption of fructose is associated with NAFLD and overconsumption of alcohol is necessary for the development of ALD, GA and AA provide a convenient link between risk factors and pathophysiology of NAFLD and ALD. For a comprehensive overview of TAGE theory, please refer to the manuscript by Takeuchi et al. [17].

Although recent advances in the understanding of the AGEs’ role in the development and progression of NAFLD and ALD are promising, AGE levels assessment and characterization are not yet incorporated into the clinical practice. Estimation of their clinical usefulness is complicated by an extensive range of studied AGE subtypes. The goal of this systematic review and meta-analysis was to evaluate if NAFLD and ALD are associated with increased levels of serum AGE. In addition, we have compared serum concentrations of AGEs and their diagnostic accuracy in patients with simple steatosis and more advanced stages of NAFLD and ALD.

## 2. Materials and Methods

The study was performed under the PRISMA guidelines [21]. The review protocol has been prospectively registered with PROSPERO, CRD42021240954 [22].

### 2.1. Search Strategy

We have searched MEDLINE, EMBASE and Cochrane Register of Controlled Trials (CENTRAL) from their inception to the final search date (4 March 2021) using terms related to liver diseases and advanced glycation end-products. In addition, we have scanned references of the full-text manuscripts. The full search strategy has been described in the Supplementary File.

### 2.2. Inclusion and Exclusion Criteria

We have included studies reporting serum concentrations of AGEs of patients with NAFLD and ALD (including simple steatosis, steatohepatitis, hepatitis and cirrhosis). The control groups were people with liver diseases excluded by normal ultrasound and aminotransferases levels. No study type restrictions were applied. We have excluded interventional studies that did not provide baseline comparison between patients with liver diseases and healthy individuals. The outcomes we sought for meta-analysis were means and standard deviations (SDs). If the publication reported medians and interquartile ranges (IQR), we checked the results for skewness [23], and if no significant skewness was found, we used the approach proposed by Shi et al. [24] and Luo et al. [25] to estimate means and SDs. If significant skewness was detected, we have reported medians and IQR obtained from the paper, without including it in the meta-analysis.

### 2.3. Assessment of Eligibility, Data Extraction, and Quality Assessment

After the removal of duplicates, two authors (KL and EW) screened abstracts and titles obtained from the search. Studies qualified after initial screening were read in full text and assessed using our exclusion and inclusion criteria. For data extraction, we have devised a datasheet with fields relating to study characteristics (title, author, study design), participants (including the type of liver disease, age, BMI, aminotransferases level) and outcomes. The risk of bias was assessed using the QUADAS-2 tool [26].

### 2.4. Synthesis of Results

Meta-analysis was conducted using the meta package [27] in R, version 4.03 [28]. Pairwise analyses were performed for each comparison where at least two studies were available. A random-effects model with the inverse-variance method was used. To detect heterogeneity, we have employed Cochran’s Q test and I^2^ inconsistency statistic. For τ^2^ we have used the Empirical Bayes estimator. Results were presented as standardized mean differences (SMD) with 95% confidence intervals. Due to a low number of papers obtained for meta-analysis, we did not perform meta-regression or sensitivity analyses.

## 3. Results

**Figure 1:**
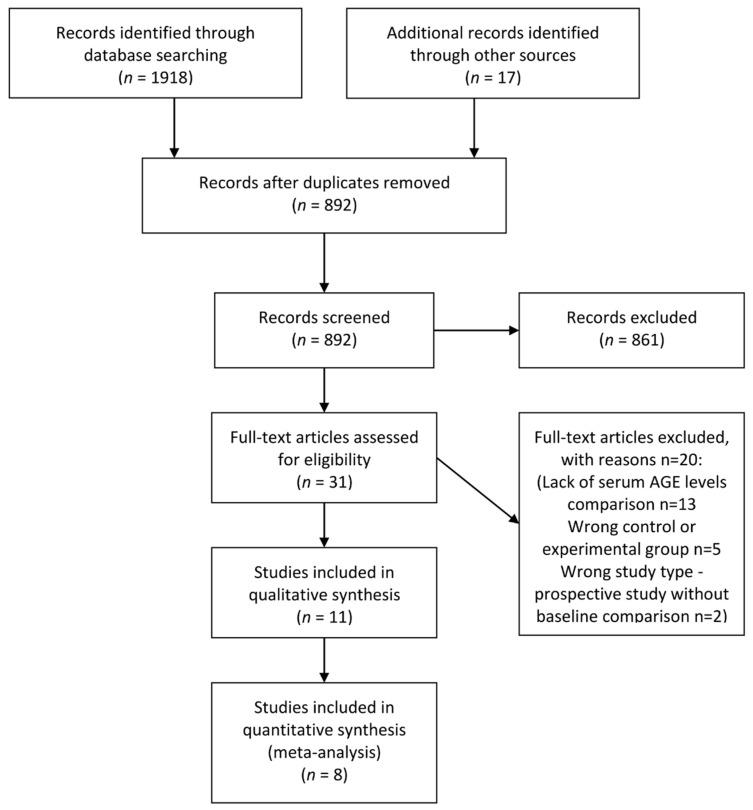
Diagram of the study selection process (adapted from PRISMA). AGE—advanced glycation end-product.

**Figure 2:**
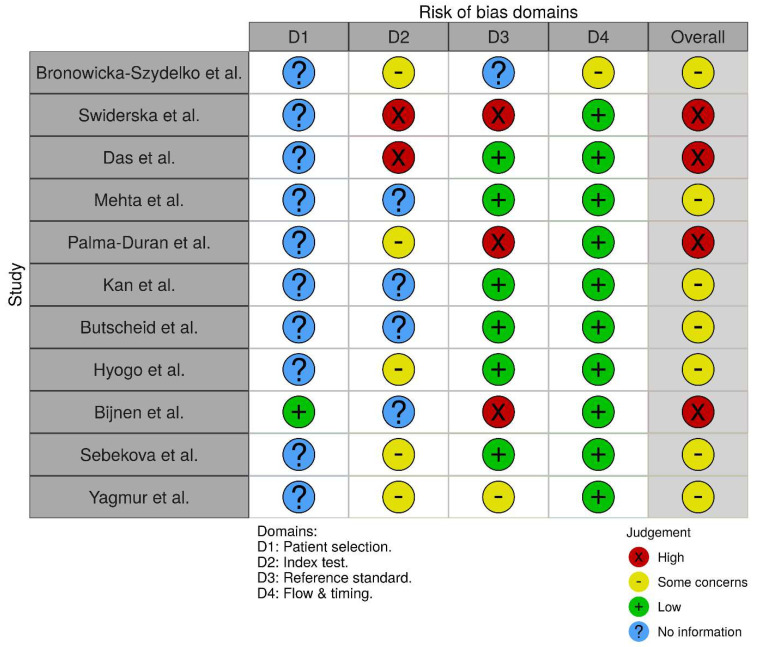
Risk of bias assessment [29,30,31,32,33,34,35,36,37,38,39].

Our search yielded 11 papers meeting our inclusion criteria, out of which eight qualified for the meta-analysis. A flowchart of the study selection process is provided in Figure 1. Obtained results and characteristics of the included studies are presented in Table 1. Seven of the included studies exhibited moderate and four high risk of bias (Figure 2). Due to significant clinical heterogeneity and a low number of obtained papers, we did not attempt to perform meta-analytical synthesis of data regarding the diagnostic accuracy of AGEs, which is instead presented as narrative synthesis.

### 3.1. Systematic Review

Studies included in the systematic review reported data on 1844 participants (909 with NAFLD, 169 with ALD and 766 healthy controls). Three studies focused on ALD [37,38,39] and eight studies on NAFLD [29,30,31,32,33,34,35,36]. In studies on ALD, the diagnosis was confirmed with a biopsy. For NAFLD, only four studies used biopsy for definitive diagnosis, the remaining four based the diagnosis on imaging (ultrasound or CT), elastography or validated equations for predicting the hepatic steatosis (using laboratory tests and other variables). Six of the studies on NAFLD reported that it is associated with higher levels of various AGEs. Out of four studies examining serum concentration of CML in NAFLD, none reported a significant difference from healthy controls. Interestingly, in the case of ALD, both studies examining CML showed that it had significantly higher serum levels in patients with alcoholic cirrhosis compared with healthy controls. One of the included studies showed, that AGE fluorescence was significantly higher in patients with advanced NAFLD (i.e., with fibrosis > 6.6 kPa measured by transient elastography) than in patients with early NAFLD (without fibrosis). 

### 3.2. Meta-Analysis

**Figure 3:**
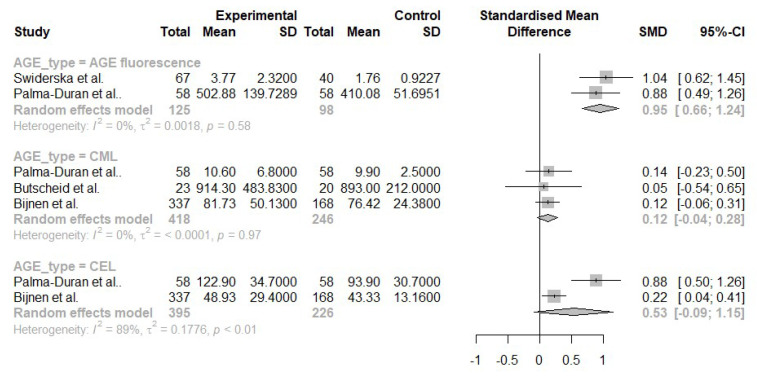
Pairwise comparisons of serum AGE levels between patients with NAFLD and healthy controls [30,32,34,36].

**Figure 4:**
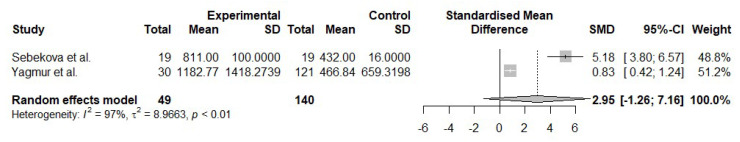
Pairwise comparisons of serum AGE (CML) levels between patients with ALD and healthy controls [38,39].

We have performed pairwise comparisons of AGE fluorescence, CML and N-(carboxyethyl)lysine (CEL) serum concentrations in NAFLD, and CML serum concentration in ALD ( Figure 3 and Figure 4).

NAFLD, in comparison to healthy controls, was associated with significantly higher AGE fluorescence (SMD 0.95, CI 0.66; 1.24) and serum concentration of CEL (SMD 0.53, CI −0.09; 1.15). CML had a negligible effect size of 0.12 with −0.04;0.28 CI. The comparison of the CEL levels was the only one with high, statistically significant heterogeneity (I^2^ = 83%, *p* < 0.01, Figure 3). 

Patients with AC had higher levels of CML compared to healthy controls (SMD 2.95, CI −1.26; 7.16). However, the comparison had very high heterogeneity (I^2^ = 97%, *p* < 0.01), which most likely stems from the extraordinarily high effect size reported by Sebekova et al. (5.18 SMD, Figure 4) [38]. The number of studies obtained for each comparison was too small to assess publication bias using statistical methods such as Egger’s test.

### 3.3. Diagnostic Accuracy

Only three studies reported data on the diagnostic accuracy of AGEs in NAFLD (Table 2). The AUC of 0.7 to 0.8 was considered acceptable, 0.8 to 0.9 excellent and >0.9 outstanding [40]. The performance of AGE fluorescence in distinguishing between low and moderate steatosis was acceptable (AUC 0.76). CML, CEL and pentosidine performed poorly in distinguishing healthy controls from patients with NAFLD; however, when coupled with sRAGE (a soluble variant of the AGE receptor), CEL/sRAGE, AGE (defined as the sum of CML, CEL, and pentosidine levels)/sRAGE and AGE fluorescence/sRAGE presented excellent discriminatory ability (AUC 0.83–0.85), exceeding the performance of both AGEs and sRAGE when used as separate markers. GA-AGE separated NAFL from NASH with acceptable performance (AUC 0.78). None of the studies with ALD patients reported diagnostic accuracy of AGEs.

## 4. Discussion

The goal of our systematic review and meta-analysis was to compare serum levels of various AGEs between patients with NAFLD or ALD and healthy controls. In addition, we have presented data regarding diagnostic accuracy in differentiating NAFLD from healthy controls and early NAFLD from more advanced stages. Types of the obtained levels of AGE compounds include CML, CEL, imidazolone, GA-AGE, glucose-AGE, AGE-1, pentosidine and AGE fluorescence.

The results of our study show that chronic liver diseases are associated with elevated concentrations of some fractions of AGEs. Interestingly, NAFLD was not associated with significant differences in serum concentrations of CML, which was frequently used as a proxy for all the subtypes of AGEs. However, the levels of CML were significantly higher in patients with AC compared with healthy controls. As we did not identify any study examining NAFLD-associated cirrhosis, we are unable to provide a definitive answer whether the difference stems from the stage (i.e., NAFL versus AC) or etiology (alcoholic versus non-alcoholic) of the liver disease. However, Yagmur et al. [39] provide some indirect evidence that cirrhosis itself—and not etiology—is responsible for higher CML levels in AC. Apart from patients with AUD, they have included other etiologies of liver injury—virus hepatitis, biliary, autoimmune and unspecified, other etiologies. They have shown that, irrespectively of etiology, cirrhotic patients had higher levels of CML than groups with chronic liver diseases but without cirrhosis. In addition, they have established a positive correlation between how advanced cirrhosis is and the concentration of CML. Unfortunately, no NAFLD group was included in that study [39]. Further evidence that CML is associated with the pathophysiology of NAFLD is provided by Gaens et al. [41]. They have shown that the accumulation of CML in the liver is significantly associated with the grade of hepatic steatosis. CEL, AGE with biochemical properties similar to CML [42] had significantly higher concentrations in patients with NAFLD than in healthy controls. Both NAFLD and ALD were associated with increased levels of AGE fluorescence.

Only one of the included studies reported the accuracy of AGEs in the diagnosis of NAFLD. None of the AGEs performed well as a sole diagnostic marker. However, when using AGEs together with sRAGE as a ratio (e.g., CEL/sRAGE), the performance significantly increased reaching an AUC of at least 0.83. This increase in the diagnostic accuracy can be explained by the physiological role of sRAGE, which serves as a receptor decoy for AGE ligand [43]. Through this mechanism, increased serum concentrations of sRAGE might lead to reduced activation of the AGE/RAGE axis, which is pivotal in the AGE-related hepatocyte injury [17].

Unfortunately, the data regarding differences in serum concentrations of AGEs in early and advanced chronic liver diseases were scarce. For ALD, the only AGE studied in this context was the fluorescent fraction, which did not differ in patients with SAH and AC. However, for NAFLD, there was a significant increase in AGE fluorescence between early (without fibrosis) and advanced disease. Additionally, AGE fluorescence distinguished low steatosis from moderate with acceptable performance. Serum concentrations of imidazolone and CML did not differ significantly between patients with NAFL and NASH. The only AGE which was significantly elevated in the patients with NASH compared with NAFL was GA-AGE. As a diagnostic marker, it had acceptable AUC (0.78), relatively high specificity (88.9%), and low sensitivity (66.7%). Therefore, our results hint that further research into different fractions of AGEs (perhaps combined with sRAGE as ratio) in various stages of NAFLD might result in clinically useful biomarkers. Out of currently available biomarkers for distinguishing NASH from simple steatosis, cytokeratin-18 is the most widely studied, with the largest trial on the topic reporting an AUC of 0.65 [44]. Distinguishing between NAFL and NASH without biopsy is currently challenging—none of the noninvasive tests have been validated for the diagnosis of NASH [4]. 

Although the exact mechanism of the development and progression of NAFLD and ALD has not yet been fully elucidated, a growing body of evidence shows that AGE might play a causative role. AGE, after binding to its specific receptor activates numerous pathways, including nuclear factor-κB (NF-κB), which is among the most widely studied compounds associated with liver injury [45]. Activation of NF-κB increases the production of proinflammatory cytokines (such as tumor necrosis factor α, interleukins 1 and 6), which has been linked with liver injury in the animal models of both NAFLD and ALD [46,47]. In addition, the binding of AGE to RAGE activates mitogen-activated protein kinases (MAPK) and c-Jun N-terminal protein kinase (JNK). The JNK/MAPK pathway is strongly associated with insulin resistance. The critical involvement of JNK1 isoform in the development of NAFLD and progression to NASH has been established using animal models. JNK1-knockout mice fed a high-fat diet had lower steatosis compared with wild type mice [48]. In the murine methionine- and choline-deficient diet model of steatohepatitis, knockout of JNK1 (but not JNK2) was associated with reduced susceptibility to NASH [49].

Apart from endogenous formation, AGEs can be provided with diet. The average daily consumption of AGE is approximately 75 mg. The main exogenous sources of AGEs include highly processed food (especially fried) and soft drinks containing high fructose corn syrup or sugar. Two main factors associated with the increased formation of dietary AGEs are high cooking temperatures and long cooking times [50]. It is not clear what fraction of dietary AGEs is absorbed into the circulation, with estimates varying between 10 and 80% [51,52]. This issue could have important practical implications—if dietary AGEs were found to significantly contribute to serum AGE levels, a low AGE diet would be a potentially viable option in reducing the severity of liver injury in NAFLD and ALD. The dietary modifications leading to the reduction of AGE content in food are relatively simple and include shorter cooking times, using lower cooking temperatures and poaching instead of frying [50]. In addition, as high pH increases the formation of dietary AGEs [53], marinating food in vinegar prior to processing might be another viable strategy. Currently, no trial examining low AGE diet in NAFLD and ALD has been performed on human subjects. However, a recent meta-analysis reported that a low AGE diet was associated with a reduction of weight and a positive impact on the markers of insulin resistance [54]. In addition, Leung et al. has shown, that diet rich in dietary AGEs was associated with increased liver injury and inflammation in an animal model of NAFLD [55].

Our study has several limitations. First, a low number of the obtained studies precluded us from performing sensitivity and more nuanced analyses such as meta-regression. In addition, for NAFLD, half of the included studies did not use a biopsy to establish the diagnosis, reducing the reliability of obtained results. Furthermore, the included papers had a cross-sectional design, which precludes making strong whether AGEs play a causative role in liver injuries. However, as we have discussed above, several studies support this notion.

Recently, another subtype has been described in human tissues, of which in vitro analogue could be synthesized in anhydrous conditions [56]. This subtype is different from glucose-related AGEs, and it is formed in physiological conditions most likely in other than known biosynthetic pathways. Our further studies will consider the relationship between its level and various clinical parameters.

In conclusion, NAFLD and ALD are associated with increased concentrations of several AGEs, and the size of the effect depends on the studied fraction. The diagnostic accuracy of AGEs is poorly studied, however the GA-AGE appears to be promising in distinguishing the patients with NAFL and NASH. Our study strengthens the link between AGEs and chronic liver diseases and shows that further research on the topic is needed and might result in novel diagnostic approaches.

## Figures and Tables

**Table 1:** Characteristics of the included studies.

Study	Study Population	Which AGE Was Measured? What Method Was Used?	How Is the Outcome Reported?	Outcome
Bronowicka-Szydelko et al. [29]	46 NAFLD 170 Control	AGE-1; Slot-blot	Median with 95% CI	Control vs NAFLD: 0 (0;9093) vs. 14580 (1049;29852); *p* < 0.05
Swiderska et al. [30]	67 NAFLD 40 Control	AGE fluorescence; Measured at 350/440 nm	Mean and SD *	Control vs NAFLD: 1.76 (0.93) vs. 3.77 (2.32); *p* < 0.001 Early NAFLD vs. advanced NAFLD: 3.36 (1.22) vs. 4.08 (1.47); *p* = 0.02
Mehta et al. [31]	103 NASH 143 NAFL 93 Control	AGE; ELISA	Mean and SD	Control vs. NAFL vs. NASH: 10.37 (4.68) vs. 9.32 (4.74) vs. 10.14 (5.31); non-significant
Palma-Duran et al. [32]	58 NAFLD 58 Control	AGE fluorescence, CML, CEL, Pentosidine; Fluorescence measured at 370/440 nm, AGEs quantification by HPLC-fluorescence-MS	Mean and SD * Median and IQR for pentosidine	Control vs NAFLD: AGE fluorescence—410.08 (51.69) vs. 502.88 (139.73); *p* < 0.001 CML—9.9 (2.5) vs. 10.6 (6.8); non-significant CEL—93.9 (30.7) vs. 122.9 (34.7); *p* < 0.001 Pentosidine—1.5 (1.4–1.7) vs. 1.6 (1.5–1.9); *p* < 0.02
Kan et al. [33]	NASH 56 Control 27	Non-CML AGE; ELISA	Mean and SD	Control vs. NASH: 3.5 (1.2) vs. 5.2 (1.7); *p* < 0.05
Butscheid et al. [34]	10 NASH 13 NAFL 20 Control	Imidazolone, CML; ELISA	Mean and SD	Control vs non-NASH NAFLD vs. NASH: Imidazolone—3.4 (1.6) vs. 3.6 (2.3) vs. 3.3 (1.4); non-significant CML—893 (212) vs. 913 (170) vs. 916 (92); non-significant
Hyogo et al. [35]	30 Control 10 NAFLD 66 NASH	GA-AGE, Glucose-AGE, CML; ELISA	Mean and SD	Control vs. NASH: Glucose-AGE—44 (22.5) vs. 72.9 (52.8); *p* < 0.005 GA-AGE—6.96 (2.36) vs. 9.78 (3.73); *p* < 0.005 CML—11.8 (7.6) vs. 16.3 (10.2); *p* = 0.054 NAFL vs. NASH: GA-AGE—7.17 (2.28) vs. 9.78 (3.73); *p* < 0.05; no glucose-AGE and CML analysis was performed for this comparison
Bijnen et al. [36]	337 NAFLD 168 Control	CML, CEL; Liquid chromatography-tandem MS	Mean and SD *	Control vs. NAFLD: CML—76.42 (24.38) vs. 81.73 (50.13); non-significant CEL—43.33 (13.16) vs. 48.93 (29.4); *p* < 0.001
Das et al. [37]	100 SAH 20 AC 20 Control	AGE fluorescence; Measured at 350/440 nm	Mean and SD	Control vs. SAH vs. AC: 8457 (2500) vs. 14574 (2670) vs. 15691 (3138); *p* < 0.01 for control vs. SAH and AC; non-significant for SAH vs. AC
Sebekova et al. [38]	19 AC 19 Control	CML; ELISA	Mean and SD	Control vs. AC: 432 (16) vs. 811 (100); *p* < 0.01
Yagmur et al. [39]	30 AC 121 Control	CML; ELISA	Mean and SD *	Control vs. AC: 466.84 (659.32) vs. 1182.77 (1418.27); *p* < 0.05

*—calculated from median and IQR. AGE—advanced glycation end-product, NAFLD—non-alcoholic fatty liver, NASH—non-alcoholic steatohepatitis, NAFL—non-alcoholic fatty-liver, SAH—severe alcoholic hepatitis, AC—alcoholic cirrhosis, CML—N-(carboxymethyl)lysine, CEL— N-(carboxyethyl)lysine, GA-AGE—glyceraldehyde-derived AGE.

**Table 2:** Diagnostic accuracy of AGEs.

Study	Comparison	Reference Test	Index Test	AUC	Sensitivity	Specificity
Swiderska et al. [30]	Low steatosis (BARD score 0–1) vs. moderate steatosis (BARD score 2–4)	BARD score	AGE fluorescence (>2.77 AFU/mg)	0.76	70%	84%
Palma-Duran et al. [32]	Healthy controls vs. NAFLD	Elevated liver enzymes, ultrasound hepatic steatosis evidence, and exclusion of other liver injuries	CML, CEL, pentosidine, CML/sRAGE	<0.78 **	NA	NA
			CEL/sRAGE (>6.9 mmol/pmol)	0.85	81%	77%
			AGE */sRAGE (>7.8 mmol/pmol)	0.85	81%	77%
			AGE fluo/sRAGE (>87.4 AU/ng)	0.83	80%	79%
Hyogo et al. [35]	NAFL vs. NASH	Biopsy	GA-AGE (>8.53/mL)	0.78	66.7%	88.9%

*—AGE represents the sum of CML, CEL, and pentosidine serum levels; **—no exact value was reported. AFU—arbitrary fluorescence unit, AUC—area under curve, NAFLD—non-alcoholic fatty liver, CML—N-(carboxymethyl)lysine, CEL—N-(carboxyethyl)lysine, GA-AGE—glyceraldehyde-derived AGE, sRAGE—soluble variant of the AGE receptor, BARD score—risk of advanced fibrosis in NAFLD.

## Data Availability

Not applicable—all data is available in primary studies.

## Supplementary Materials

The following are available online at https://www.mdpi.com/article/10.3390/nu13103370/s1: Supplementary File, Search Strategy.
