# Supplementary material for: Advanced Glycation End-Products in Common Non-Infectious Liver Diseases: Systematic Review and Meta-Analysis
Source: Nutrients. 2021 Sep 25;13(10):3370. doi: 10.3390/nu13103370 (PMC8537188; doi:10.3390/nu13103370)
Supplement: Supplementary file 1 [file nutrients-13-03370-s001.zip › nutrients-1373088-supplementary.pdf]

PUBMED:

("advanced glycation end product*" OR "advanced glycation endproduct*" OR "advanced glycation end-product*" OR "Glycation End Products, Advanced"[MeSH] OR imidazolone OR (CML NOT "chronic myeloid leukemia") OR RAGE OR AGER OR "Receptor for Advanced Glycation End Products"[MeSH])

AND

(("fatty liver"[MeSH] OR hepatitis[MeSH] OR "liver cirrhosis"[MeSH] OR steatosis OR steatohepatitis OR "nonalcoholic steatohepatitis" OR "non-alcoholic steatohepatitis" OR NAFLD OR NASH OR "fatty liver disease" OR FLD)

OR

("alcoholic steatohepatitis" OR "alcoholism"[MeSH] OR "Liver Diseases, Alcoholic"[MeSH] OR ALD OR ASH OR "alcoholic steatohepatitis" OR "alcoholic hepatitis"))

EMBASE:

("advanced glycation end product*" OR "advanced glycation endproduct*" OR "advanced glycation end-product*" OR 'Glycation End Products, Advanced'/exp OR imidazolone OR (CML NOT "chronic myeloid leukemia") OR RAGE OR AGER OR 'Receptor for Advanced Glycation End Products '/exp)

AND

(('fatty liver'/exp OR 'hepatitis'/exp OR 'liver cirrhosis'/exp OR steatosis OR steatohepatitis OR "nonalcoholic steatohepatitis" OR "non-alcoholic steatohepatitis" OR NAFLD OR NASH OR "fatty liver disease" OR FLD)

OR

("alcoholic steatohepatitis" OR 'alcoholism'/exp OR 'Liver Diseases, Alcoholic'/exp OR ALD OR ASH OR "alcoholic steatohepatitis" OR "alcoholic hepatitis"))

CENTRAL:

#1 ("advanced glycation end product*" OR "advanced glycation endproduct*" OR "advanced glycation end-product*" OR imidazolone OR (CML NOT "chronic myeloid leukemia")) (Word variations have been searched)

#2 MeSH descriptor: [Glycation End Products, Advanced] explode all trees

#3 RAGE OR AGER

#4 MeSH descriptor: [Receptor for Advanced Glycation End Products] explode all trees

#5 #1 OR #2 OR #3 OR #4

#6 MeSH descriptor: [Fatty Liver] explode all trees

#7 MeSH descriptor: [Hepatitis] explode all trees

#8 MeSH descriptor: [Liver Cirrhosis] explode all trees

#9 steatosis OR steatohepatitis OR "nonalcoholic steatohepatitis" OR "non-alcoholic steatohepatitis" OR NAFLD OR NASH OR "fatty liver disease" OR FLD

#10 #6 OR #7 OR #8 OR #9

#11 MeSH descriptor: [Alcoholism] explode all trees

#12 MeSH descriptor: [Liver Diseases, Alcoholic] explode all trees

#13 "alcoholic steatohepatitis" OR ALD OR ASH OR "alcoholic steatohepatitis" OR "alcoholic hepatitis"

#14 #11 OR #12 OR #13

#15 #5 AND (#10 OR #14)
